# An Address to the Kensington Branch of the League of Nations Union

**Published:** 1921-04-30

**Authors:** William J. Collins


### SOME INTERNATIONAL HEALTH QUESTIONS AND THE LEAGUE OF NATIONS.

### An Address to the Kensington Branch of the League of Nations Union.

By Sir WILLIAM J. COLLINS, K.C.V.O., M.S., M.D., B.Sc. (Lond.), F.R.C.S. (Eng.).

Public health, as we usually employ that term, connotes a conception and a policy which are of relatively recent development in the modern world. When Queen Victoria came to the throne in 1837 the Statute Book of this country contained no general law of sanitary intention, unless the antiquated Quarantine Laws and a Parliamentary annual grant to the National Vaccine Board can be so classed. "Outside these two matters," according to the late Sir John Simon, "the Central Government had nothing to say in regard to the public health, and local authorities had but the most indefinite relation to it. Various important towns had their special Improvement Acts for certain purposes, but among these purposes health had hardly yet begun to stand on its own merits."

Take the question of Housing, so proximately related to the health of the people, and now so prominent in the public eye, yet it did not engage the attention of our Legislature or local authorities prior to the middle of last century, unless, indeed, we recall the 35th of Elizabeth, chapter 6, which was only repealed in 1888, and which forbad the erection of additional houses in London and Westminster, lest those cities should become larger than was deemed compatible with those "spacious days." Even in 1855, when London beyond the City was first endowed with some semblance of a central municipal authority in the shape of the late lamented Metropolitan Board of Works, no powers in respect of housing the working classes were conferred by the Legislature on that body.

### The Modern Trend.

Since that time, not only here but in all civilised communities, the trend has been increasingly manifested for States and Municipalities to concern themselves with circumstances related in various ways and degrees to the health of their peoples. As in so many other directions, duties formerly either by intention or neglect reposed in private hands or left to voluntary agency or individual initiative, have passed into the province of the local authority or the State, and not a few of these have engaged or are now engaging *international* consideration and co-operation by way of diplomatic conferences, international conventions, and congresses. A regard for first principles might invite an interesting discussion on the problem as to what duties or rights in regard to health-promotion or disease-prevention should be allocated respectively to individual, local, national, or international jurisdiction. John Stuart Mill and Mr. Herbert Spencer are now irreverently dismissed as "back numbers" by the dispraisers of the time gone by, and despisers of the Victorian age, but it may be worth recalling that the former, in his "Essay on Liberty," maintained that "Each is the proper guardian of his own health, whether bodily or mental and spiritual. . . . Over himself, over his own body and mind the individual is sovereign." So also Wilhelm von Humboldt, in prescribing "the sphere and duties of Government," went so far as to insist that "the State is to abstain from all solicitude for the positive welfare of the citizens, and not to proceed further than is necessary for their mutual security and protection against foreign enemies; for with no other object should it impose restrictions on freedom." Baron von Humboldt also vouchsafed the opinion that "national education" seemed to him to "lie wholly beyond the limits within which political agency should properly be confined," and then subsequently falsified his doctrine by accepting the position of Minister of Public Instruction in Prussia. Such teaching as that of Mill and Humboldt sounds very out-of-date to-day, when a benevolent, if despotic, philanthropy, inspired by eugenic philosophy, is inviting the aid of State medicine in supervising the health of the individual from the ante-natal clinic, through the seven ages of man, to the grave or the crematorium.

### Sanitary Reform.

The modern demand for sanitary reform—for the application of improving knowledge as to the preventability of disease, and the potent influence of environment in the promotion or impairment of health, may be traced to the fourth decade of last century, and in this country more especially to Sir Edwin Chadwick, his co-workers, and those who shared his school of thought, under the continuing inspiration of that "new humanity," which had set Howard to reform our prisons, Wilberforce to enfranchise the slave, and Romilly to ameliorate our barbarous criminal code. Doubtless there were many illustrious obscure whose names are not recorded, or are forgotten, who did similar work, and were sustained by similar high ideals to those whom I have named. Something was due to the spirit of the times, for not only here but on the Continent the nineteenth century, before it had sounded its jubilee, had witnessed an outburst of sanitary reform which was awakening or re-awakening a gospel of cleanliness, long overlaid or obscured by superstition, formalism, indifference, or self-satisfied ignorance.

This humanitarian zeal, reinforced by a more scientific pathology and penology, was powerfully fomented by the writings of Jeremy Bentham, the great utilitarian philosopher. It is well to recall that it is to him we are indebted for coining the word "International," now so frequently on our lips, and he sought to apply his principle of "the greatest happiness of the greatest number" not only nationally, but internationally. Bentham's long life extended from 1748 to 1832. In earlier years he was the friend of the Wesleys, Adam Smith, Pitt, and Mirabeau; in middle life the elder Mill and Romilly were his intimates, while Chadwick lived with him in his later years in a secretarial capacity, and imbibed from the master much of that zeal for reform which inspired his labours for the betterment of the public health. There is a reciprocity, a pre-established harmony, as it were, between "internationalism" and "idealism." Humanitarianism naturally tends to expand itself beyond the narrower limits of patriotism, and embraces all mankind. So also the contemplation of the international aspect of humanitarian questions such as slavery, the treatment and the lot of Labour, the repression of zymotic diseases, or the traffic in noxious drugs, lifts political action to a higher plane, gives a loftier as well as a wider outlook on human endeavour and the sickness and suffering of mankind, while it imparts an altruism to international effort, reminding us once again that "patriotism alone is not enough."

### Humanitarian Questions.

The Covenant of the League of Nations and the Peace Treaties with Germany, Austria, Bulgaria, Hungary, and Turkey conventionalise and internationalise many questions touching directly or indirectly the physical health and moral welfare of mankind. Thus Article 23 (*a*) seeks to secure "fair and humane conditions of labour for men, women, and children"; 23 (*c*) entrusts the League with "the general supervision over the execution of agreements with regard to the traffic in women and children and the traffic in opium and other dangerous drugs"; 23 (*f*) binds the League to endeavour to take steps in matters of international concern for the prevention and control of disease; Article 25 records the agreement of members of the League "to encourage and promote the establishment and co-operation of duly authorised voluntary national Red Cross organisations having as purposes the improvement of

**Some International Health Questions—(*continued*).**

health, the prevention of disease, and the mitigation of suffering throughout the world." The wording of that article is significant as indicating that the good offices of the League may be available for facilitating international co-operation on the part of voluntary organisations, of *assistance privée* as well as *publique*, as the French would say, and have regard to philanthropic endeavours outside the limits of international Conventions or municipal laws.

The Covenant also touches, though less directly, health questions in Article 22 in providing for the tutelage of "peoples not yet able to stand by themselves under the strenuous conditions of the modern world" and "the prohibition of such abuses as the slave trade . . . and the liquor traffic." Moreover, the Treaty of Versailles and the other treaties which followed it make provision for carrying on certain pre-existent agreements with the late enemy Powers in matters of health, and prescribe the machinery for the future application of such agreements, and of others that may become the subject of international Conventions.

Thus Article 282 of the Versailles Treaty requires the application as between the Allied and Associated Powers and Germany of the Sanitary Conventions of 1892, 1893, 1894, 1897, and 1903, of the 1906 Convention for the suppression of the use of white phosphorus in the manufacture of matches, of the 1906 Convention for the suppression of night work for women, and of the 1906 Convention for the unification of pharmacopœial formulæ for potent drugs. Article 295 of the same treaty deals with the effectuation of the Opium Convention of 1912, which the enemy Powers had previously failed to ratify. Article 285 (2) refers to the North Sea liquor traffic, while Part XIII., comprising Articles 387-427 of the Treaty, provides for the procedure and principles to be observed in the organisation of Labour under healthier and humaner conditions.

I must also refer to Article 24 of the Covenant, which is very important, somewhat indefinite, but may have far-reaching effect on the relations of many international co-operative efforts to the League of Nations and its secretariate, especially those of a hygienic or philanthropic character.

It provides for placing under the direction of the League (with the consent of the parties concerned) of all international bureaus already established by general treaties. All such bureaus henceforth established shall be under the League's direction. All matters of international interest regulated by general conventions, but not under international bureaus or commissions, *may* by mutual consent be assisted by the League, which will collect and distribute relevant information. Thus the Sanitary Conventions, notably that of 1903, directed against the spread of cholera, plague, and yellow fever, and other pre-existent health treaties, may this way pass under the League's direction, as well as those new duties for which the League assumes responsibility in Articles 22, 23, and 25, to which I have already alluded.

From a discussion, in which I took part, at the International Law Association at Portsmouth last May, and from communications I have had with the Secretariate of the League of Nations, I gather that the latter is disposed to take a wide view of its responsibilities in relation to international co-operation and organisations under Article 24 of the Covenant.

The Secretariate is, I gather, ready to cast its ægis over voluntary associations of an international character with scientific, juridical, or sociological objectives, even if they have not been the subject of diplomatic conferences or international conventions. The case of the Congress of Social Work and Service came under special consideration, since the war interrupted the holding of its sixth session in London in 1915, for which arrangements had been made and subscriptions from various nationalities received. In this case a small portion only of the work of the Congress had been the subject of an international convention, but the League is apparently not indisposed to assume direction thereof under Article 24 if requested to do so. In this case, as in several others, there is also the complication arising from the fact that the League is not as yet so widely international as were some of these voluntary organisations prior to the war.

Scientific, social, and sanitary problems demand consideration in the widest international sense, and would be very inadequately envisaged if we omitted the United States, Russia, or Germany and the other late enemy Powers from the scope of our purview.

Take the case of epidemic disease, which laughs at scientific frontiers and geographical expressions, and pays little regard to nationality or ethnographical delimitations. Though we may affect to speak of the Asiatic cholera, the Levantine plague, the Spanish influenza, the German measles, and the French disease, we merely thereby chronicle a sorry nomenclature wherewith a short-sighted epidemiology seeks to shift responsibility and foist upon another people the causation of pestilence for which home neglect has too often provided a breeding-ground or at least a fertile soil for the propagation of disease.

Having briefly referred to the powers set out in the Covenant of the League of Nations which touch directly or indirectly on international co-operation on matters relating to health and the prevention of sickness and suffering, let us inquire what action has thus far been taken, under the auspices of the League, by way of preparing the requisite organisation for carrying out the humanitarian objects in view, as well as any by way of direct operations to meet immediate necessities.

In the Report by the Secretary-General of the League of Nations (Sir Eric Drummond) on the work of its Council, submitted to the first assembly of the League on November 15, 1920, at Geneva, health questions do not bulk very large. A special report is referred to on "The institution of a Permanent Health Office" (p 19), and under the heading "Action of the League in the general interests of humanity" the campaign against typhus in Poland is dealt with. This serious menace to the health of Europe appears to have engaged the attention of the Council at its second, third, seventh, eighth, and tenth sessions in London, Paris, San Sebastian, and Brussels between February and October of this year. An International Health Conference which met in London in April 1920 was invited to submit plans for united official action against the westward spread of typhus. As the result the Council sent an Executive Commission to Poland to effect the co-operation of the Polish Health Authorities with the Red Cross Societies. Mr. Balfour was asked to appeal to the twenty-eight member States for a sum of £2,000,000, and £250,000 for immediate use. The response was reported to be disappointing; many of the Governments appealed to had vouchsafed no reply. Great Britain and France had made conditional offers of £50,000, while Canada, Greece, Persia, Belgium, and Siam also offered contributions. Those who heard Dr. White's Chadwick lecture on July 15, 1920, on his experiences in Eastern Europe realised the truth of Hirsch's statement in his "Handbook of Geographical and Historical Pathology"—that the "history of typhus is written in those dark pages of the world's story which tell of the grievous visitations of mankind by war, famine, and misery of every sort."

(*To be continued.*)

